# Experiences in the Decision-Making Regarding the Place of Care of the Elderly: A Systematic Review

**DOI:** 10.3390/bs11020014

**Published:** 2021-01-21

**Authors:** Gema Serrano-Gemes, Rafael Serrano-del-Rosal, Manuel Rich-Ruiz

**Affiliations:** 1 Institute for Advanced Social Studies- Spanish National Research Council (IESA-CSIC), 14004 Cordoba, Spain; rserrano@iesa.csic.es; 2 Maimonides Biomedical Research Institute of Cordoba (IMIBIC), University of Cordoba (UCO), Reina Sofia University Hospital (HURS), 14004 Cordoba, Spain; en1rirum@uco.es; 3 CIBER on Frailty and Healthy Ageing (CIBERFES), 28029 Madrid, Spain

**Keywords:** decision-making, place of care, location of care, elders, aged, experiences, systematic review

## Abstract

The objective of this review was to understand how participants experience the decision-making process regarding the place of care for the elderly. Therefore, we conducted a systematic review of qualitative studies. The articles were included if they were original studies with qualitative/mixed methodology, written in English/Spanish, and that approached the decision-making process regarding the place of care for the elderly, already experienced by the participants. Forty-four articles were included, identifying experiences, both negative and positive. Negative experiences have been the most frequently reported experiences by all population groups; fear was the most relevant experience for the elderly, whereas concern was the most relevant for family members and professionals. This review has not only found a great variability of experiences, but also, it has deepened the differences between groups and the situations motivating/generating these experiences. This review highlights a wide range of experiences of those directly involved in the entire decision-making process on the place of care for the elderly. In future research it would be interesting to carry out qualitative primary studies conducted with professionals and other relevant people involved in this decision-making process, in order to know first-hand how they experience this process.

## 1. Introduction

The perspective of the United Nations indicates that the worldwide population continues to increase today, although at a slower pace, with an expectation that it will continue growing in the coming years, reaching 8.5 billion people by 2030, 9.7 billion by 2050, and 10.9 billion by 2100 [1]. The same report also estimates that there will be a worldwide increase in life expectancy at birth, from 72.6 years in 2019, to 77.1 years in 2050 [1].

Thus, the United Nations indicates that, traditionally, the ageing of the population occurs due to an increase in longevity and decrease in fertility [1]. Anticipating the aforementioned report, the proportion of older people worldwide will reach almost 12% by 2030, 16% by 2050, and 23% by 2100 [1].

This is important, because, as the World Health Organization points out in the World Report on Ageing and Health, care dependence increases as age increases, which consequently will cause the ageing of the population to lead to a significant increase in the number of people requiring social assistance, which will affect countries of all levels of development [2]. A few years ago, in a report on long-term care, the United States Department of Health and Human Services reported that the demand for long-term care for the elderly, whether in community or institutional care settings, was expected to increase [3].

However, most health systems are not ready to offer the comprehensive care necessary to be able to treat the multimorbidity of the elderly [2]. In addition, the different needs and personal preferences of older people who have functional problems and chronic diseases are not considered [4].

In this sense, the elderly usually prefer to stay in their local community, being able, whenever possible, to maintain their social networks [2], an aspect that is supported by the United Nations in its principles for the elderly, according to which, the elderly must be able to live in environments that are not only safe, but also adaptable to their individual preferences and to their changing capacities, with it also being important that the elders can live at home for as long as possible [5].

However, one should bear in mind that long-term care for the elderly can be offered in a multitude of settings and in all of them the intensity and scope of the provided support and care can differ [2]. These places can be their own home, nursing homes, assisted living facilities, hospitals, community centers, and even other health facilities [2].

Therefore, due to the problems described above, and the diversity of existing care options, making a decision regarding the place of care for the elderly becomes necessary.

In general, as Schumacher and Meleis (2009) [6] point out, transitions have become a topic of great interest for nursing due to their effects on health. Thus, these authors highlight four types of transition, within which there are different subtypes, such as transitions between different levels of healthcare within the health system (throughout the course of a disease), as well as changes in family conditions, being the transition of an older person from home to a nursing home, an example studied by different authors throughout the literature as noted by Schumacher and Meleis (2009) [6] in their review [6].

The aforementioned authors also point out that transitions are linked to a wide variety of emotions, which, normally, are responsible for showing the different problems arising throughout the transition. The understanding of the transition from the point of view of the people who experience it is essential [6].

In fact, regarding residential decision making, Hays (2002) [7] indicates in her review of the literature that the evidence suggests that older people often have past experiences and ideas of with whom they plan to live and where they will do so. However, this author points out that sometimes these personal preferences and opinions are not considered in clinical planning or evaluations, which can cause nurses to make mistakes regarding patient preferences and knowledge about a patients’ options. This review also notes that when the conditions of life preferred by the patient are not achieved, some may feel grief or regret, thus being affected in an important way their emotional quality of life [7].

Furthermore, Oswald and Rowles (2017) [8] note that in order to fully address the relocation decision, it is necessary to study and contrast the way that people make this decision, that is, it is necessary to compare those who decide to relocate with those who, despite the family environment potentially being an inappropriate setting, decide to continue living in it and not relocate. However, these authors reported that, despite the benefits and opportunities that this type of study would bring (such as a greater understanding of the decision-making process, the comparison of risk factors, and an increase in knowledge about the resources used to make decisions), only a few studies have taken into account these two groups [8].

Finally, the literature highlights that decision making may be influenced by both positive and negative emotions [9]. More research is needed on how older people make health decisions [10]. Due to all of the factors mentioned above, our aim was to understand how participants experience the decision-making process regarding the place of care of the elderly.

It is necessary to mention that this review is part of a broader review on this topic, which focuses on three main aspects: “participants”, “experiences”, and “reasons”. The protocol for this broader systematic review was recorded in PROSPERO (registration number: CRD42018084826), and subsequently published [11]. Regarding the first of the three aspects, that is, the participants, a study approaching this thoroughly has been published recently [12]. In relation to the third of the three aspects, that is, the reasons, a study addressing this issue in depth has recently been published [13]. Therefore, the present study deals with the second of these three aspects.

## 2. Methods

### 2.1. Design

A systematic review of qualitative studies was performed.

This review has been written following the Enhancing transparency in reporting the synthesis of qualitative research (ENTREQ) statement [14] and the Preferred Reporting Items for Systematic Reviews and Meta-Analyses (PRISMA) statement [15] (see Supplementary Materials file 1, PRISMA Checklist).

### 2.2. Sampling

Systematic searches were conducted in several databases of interest: CINAHL Complete (through EBSCOhost), MEDLINE (through PubMed), PsycInfo (through ProQUEST), SciELO Citation Index (through Web of Science), Scopus and Web of Science (core collection of Web of Science).

The search strategies were made up of five key concepts: four focused on elements of interest and a fifth concept focused on aspects that were far from the objective of the review. These concepts were: (1) the elderly; (2) the place of care/relocation; (3) different generic options of places of care; (4) decision making; and (5) elements far from the main objective of the review: substance abuse, intellectual disability, place to die, the end of life, palliative care, advance directives, advance care planning, and terminal patient care. Each of these concepts was, in turn, composed of multiple specific search terms.

The terms made reference to each concept and were connected using “OR”. Later, the concepts 1, 2, 3, and 4 were connected using “AND”; meanwhile, “NOT” was used to connect the fifth one with the other four concepts.

In order to perform the search in the different databases of interest, it was necessary to adapt the search strategy to each database, although always maintaining the structure and key concepts. The specific search strategies used, which were adapted to each database, can be consulted in the systematic review protocol [11]. The searches identified relevant articles from the outset until 29 November 2017.

Besides, and in order to ensure that we gathered all the important literature on this subject, the reference list of the articles finally included in this review was also reviewed.

### 2.3. Inclusion/Exclusion Criteria

The articles eligible to be included in this review were: original studies with qualitative or mixed-methods, written in Spanish or English, and dealing with the decision-making process on the place of care of the elderly (persons aged 65 or above) already experienced by the participants.

However, the articles excluded were those dealing with other decisions, such as: deciding about the place to die, advanced directives, terminal patient care, advanced care planning, palliative care, and/or the end of life; temporary locations of care, specific health problems (such as psychiatric inpatient care), and/or decisions on acute care; decisions on the place of care linked to intellectual disabilities or substance abuse. Moreover, studies where relocation started in an institutional environment were excluded. Studies that did not specifically address the main objective of this review were also excluded. Finally, studies whose complete text was not accessible, as well as doctoral theses and conference proceedings (conference abstracts), were excluded too. 

More details about the inclusion/exclusion criteria can be consulted in the review protocol [11].

### 2.4. Data Collection Process

**Figure 1:**
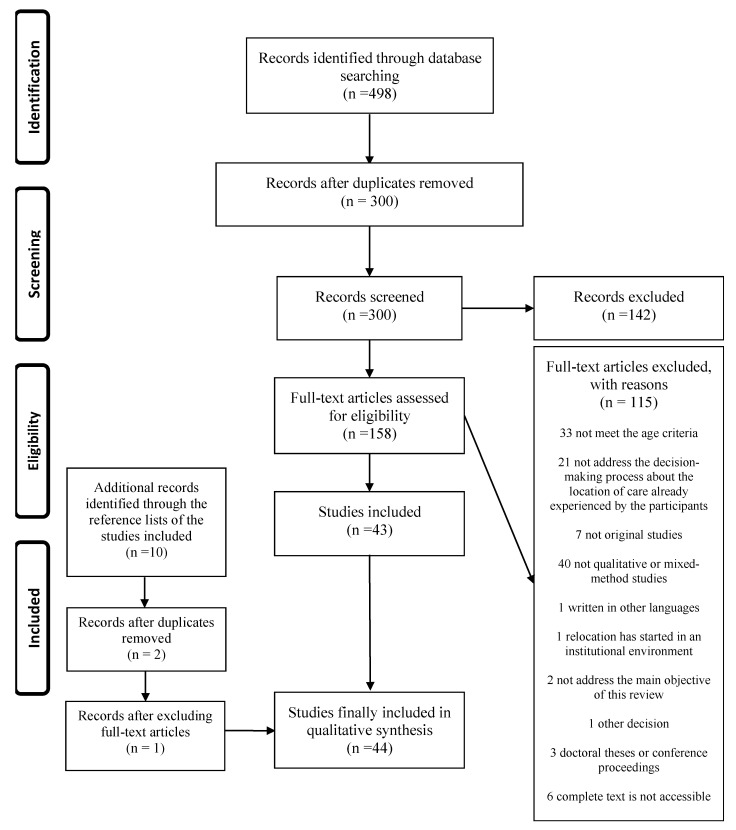
Modification of the Preferred Reporting Items for Systematic Reviews and Meta-Analyses (PRISMA) 2009 flow diagram [15]. Source: own elaboration following the information obtained. (More detailed information about “the identification” can be found in Serrano-Gemes et al. [11]).

The studies obtained by our search strategy were screened by tittle and abstract, and then, the full-texts were reviewed. This process was performed by two reviewers, a third reviewer helped them when needed. Figure 1 shows a flow diagram of this process.

### 2.5. Data Abstraction

Two authors were independently responsible for extracting the relevant information from the studies finally included in the review, asking a third author when needed. A tool designed for this purpose was used, information regarding the descriptive aspects of the studies and the aspects of interest related to the objective of the review was collected using this tool. This tool was piloted with a sample of four articles to verify that its functioning was correct.

### 2.6. Synthesis

The method of analysis used was the constant comparative method [16] from Glaser and Strauss’ grounded theory [17]. Two authors read the articles included in the systematic review several times in order to understand the studies in depth. At the same time, they identified the information related to the objective of the study. Later these authors compared the information applicable to each category and classified the information of interest, obtaining different categories and subcategories, asking the third author in case of disagreement. Finally, all authors reviewed, checked, and discussed the final results in order to guarantee that they suit the original information and the internal coherence of the results.

According to Lockwood et al. [18], it is important to note that throughout the entire analysis process, the identification of information of interest did not focus solely on the similarity in wording, but, due to the conceptual complexity of many terms, key concepts related to the objective of the review were also identified, which, as described in Noblit and Hare’s method, can always be expressed with the same words or with different ones [19]. Thus, as a result of repeated reading and a detailed analysis process, new knowledge has been created, which, as the aforementioned authors point out, deepens and goes beyond the content of the original studies [19].

### 2.7. Quality Appraisal

The quality assessment was conducted with the studies included in this systematic review. This evaluation was developed by two authors, with the third author mediating only in cases of disagreement. For this purpose, a template to help understand a qualitative study designed by the Critical Appraisal Skills Programme Español (CASPe) [20] was used. This tool is composed of 10 questions, which are intended to help assess the quality of qualitative studies. These questions have three answer options: “YES”, “NO”, and “NOT SURE” [20].

To rate the quality levels of the studies included, we used the classification suggested by Butler et al. [21]. This categorization scores the “YES” response with 1 points, the “NOT SURE” with 0.5, and the “NO” with 0. Subsequently, these authors classified these scores into four categories: scores of high-quality (9–10), scores of moderate-quality (7.5–9), scores of low-quality (less than 7.5), excluding those studies with less than 6 points [21]. However, in our review, only the first three categories have been used, including in the low-quality category all those studies scoring below 7.5 points and no studies have been excluded based on their quality.

In addition, and with the objective of performing some kind of sensitivity analysis [22], a study of the relative contribution of the studies according to their quality has been performed, considering a process previously suggested by other authors [19]. Thus, this review has considered the information provided by the different studies to the results of our review as a relative contribution. Therefore, when a study is scored with a relative contribution, for example, of 5, it means that this study has provided information to the results on 5 occasions.

## 3. Results

### 3.1. Search Results

This systematic review finally included forty-four studies (published in English). Regarding the participants of the included studies, the participants were older people in 27 studies, 22 studies included relatives, 5 studies included healthcare professionals, 2 studies focused on the personal experience of the researcher as a relative of an elder, and 1 study used patient records. These studies were mainly conducted in the United States (18) (see Supplementary Materials file 2, “Characteristics of the included studies”).

In summary, in relation to the methodological quality of the included studies, 8 out of the 44 articles included have had a low-quality (L), 15 a high-quality (H), and 21 a moderate-quality (M). Furthermore, there seems to be no relationship between the relative contribution of the included studies to the results obtained and the quality of the studies. This occurs because there are some articles that despite having a low contribution to the review show high quality, at the same time some studies with a large contribution showed low quality (see Supplementary Materials file 2, “Characteristics of the included studies”).

### 3.2. Synthesis Results

The main result of our review is the collection of information regarding a wide range of experiences occurring throughout the entire decision-making process. These experiences can be positive and/or negative, they occur in different population groups, and they may or may not coincide with each other, both in the experiences themselves and in how they originate.

Therefore, for the purpose of clarity, this section of results has been divided into two main sections: experiences and differences between groups; the first of these being subdivided, in turn, according to the different groups of participants found, following the classification proposed in another recently published article: the elderly, family members, healthcare and social service professionals, and other relevant participants [12].

Participants have used various methods to express their experiences in relation to this process, whether that be thoughts, memories, desires, valuations, emotions, feelings, sensations, and even actions and behaviors. Therefore, throughout this review, the concept “experience” will be used to combine all these terms, which, in one way or another, describe how the participants lived and experienced this process.

Finally, a relationship has also been established between the reported experiences and the situations in which they were referred, in an attempt to describe and understand the experience in greater depth and detail. This information, in conjunction with some examples of quotations, are shown in Table 1, Table 2, Table 3, Table 4 and Table 5.

### 3.3. Experiences

- The elderly

Older people refer to various experiences, both positive and negative, which have been classified in turn, in a wide variety of subcategories. The positive experiences are composed of 28 subcategories, while the negative subcategories are almost double this number, giving a total of 61 different subcategories. All of these subcategories are shown in the Supplementary Materials file 3, “List of experiences”, ordered from highest to lowest frequency of mention. Furthermore, Table 1 and Table 2 show the most frequently reported experiences (positive and negative, respectively) and the situations to which they refer. Original quotations (Q) can be found in the Supplementary Materials file 4, “Original Quotations”.

- Family members

Similar to the older people, family members report both positive and negative experiences, the latter being much more abundant, in both quantity and variety. Thus, 31 positive subcategories were obtained in contrast with 63 negative subcategories. The full list of experiences (positive and negative), ordered from the highest to lowest frequency of reference, can be found in Supplementary Materials file 3, “List of experiences”. In turn, Table 3 and Table 4 show the different situations in which the most frequently reported experiences (positive and negative) are referred; some quotations obtained can be found in Supplementary Materials file 4, “Original Quotations”.

- Professionals

Despite not having found many studies addressing the experiences of healthcare and social service professionals who are involved in the decision-making process, positive experiences [54,58,61] and negative experiences [34,36,47,54,58,66] have been found; obtaining thus 3 positive subcategories: satisfaction [58], successful experience [61], and being neutral and non-directive [54]; and 7 negative subcategories: frustration [58], ethical dilemmas [58], reluctance [34], conflict [34], tension [34], fear [34], and concern (the most mentioned) [34,36,47,54,58,66].

- Other relevant participants

Despite being a population group reported in different studies as involved in the decision-making process [12], no mention of positive or negative experiences has been found.

### 3.4. Differences between Groups

Another of the most relevant results of this study has been the discovery of exclusive experiences for each population group. These are experiences that, despite their importance, are not found in the other groups analyzed. Table 5 shows some examples of the most important experiences.

Furthermore, another relevant aspect found in this review has been the discovery of experiences that, despite being reported by the different population groups, are sometimes very different regarding their content, the situations to which they refer, or the reasons that cause them.

This occurs, for example, in the case of fear, concern, difficulty, or feeling of conflict.

Fear and concern were highlighted as extremely negative experiences, both for the elderly people and for family members and professionals. However, the motivations were different for the various groups, and the frequency with which it was reported was also different.

Fear was the most relevant negative experience for the elderly, while concern was the most relevant for family members and professionals.

Regarding concern, despite agreeing in multiple aspects in a general way, such as the concern for costs, the safety of the elderly, for carers/family members, etc., there are certain differences, the concern for the opinions that others may have being one of the most relevant, which only affects family members.

A similar situation occurs with fear, family members fear the opinions of others, lack of information or lack of control, while older people fear relocation, the future, the burden on their family, or the loss of autonomy/fear of dependence.

In the case of difficulty, this was reported by the elderly and family members as being an important negative experience. However, despite having many similarities, family members focus on the difficulties more related to care, to practical organizational aspects, to the health system or to the difficulties surrounding the decision making itself, while older people focus more on the time of relocation, what relocation implies, or the stigmas associated with age.

Finally, although the reasons that lead to the feeling of conflict is quite consistent between the groups, the frequency with which it is reported does not coincide amongst the elderly, professionals, and family members. Conflict is among the most negative experiences only for family members.

## 4. Discussion

The most important result of this systematic review of qualitative studies is the identification of a large number of experiences that occur during the entire decision-making process involved in deciding the place of care in old age and in the different population groups involved.

These experiences are both positive and negative. Negative experiences are much more predominant, not only in quantity, but also in variability.

Regarding the different population groups involved in the process, the elderly and family members are those who have contributed a larger number of experiences in relation to this process, followed by the professionals, with much less. In contrast, the other relevant group has not contributed any experience in relation to this process.

Within the group of the elderly, our results have shown how they experience more negative experiences than positive ones. This is consistent with a review of the literature on the experiences of older people with residential care placement, which states that it is not uncommon that less positive than negative experiences are identified, because residential care placement for the elderly is a fact that is often associated, quite frequently, with crisis [67].

Among the negative experiences, fear appears in our study as the negative experience most often reported by older people, reflected by multiple situations, such as fear of isolation/loneliness. This aspect is supported by a review of the previously published literature [68], which is consistent in a certain way with our results, since they mention that older people are afraid of being alone because of the insecurity of not knowing if the caregiver will go or not.

However, our results also show how older people experience positive experiences, which again is consistent with the report by Lee et al. [67], who reported that there are older people who make a positive description of their experiences, expressing usually feelings of safety and relief.

Therefore, relocation is not always seen negatively, as older people even mention that they crave for the location. This aspect of our results seems to be consistent with a quantitative study about the wellbeing of women after relocating to independent living communities, which shows how the total quality of life index score improved significantly for women after moving, with most of the participants feeling a greater satisfaction of life in general [69].

However, in contrast with our results showing previous experience as a positive aspect for the elderly, a study conducted by Maust et al. [70] shows that prior experience helping make decisions in the field of health for other adults as surrogates did not influence treatment decisions.

Regarding carers, the systematic review of the literature of Jacobson et al. [71], highlights how carers experience a mixture of different feelings simultaneously, including sadness, guilt, loss of control, and even relief. Which, in a certain way, is consistent with our results, because the relatives and the elderly reported both positive and negative experiences, the latter being much more abundant in both groups.

Regarding the positive experiences most often reported by family members, our results find both consistencies and inconsistencies with other systematic reviews of the literature on the subject. Specifically, our results highlight the importance of professional and informal support, and the feeling of relief as part of the positive experiences of family members. The review of Jacobson et al. [71] is consistent in a certain way with our results, because it describes that one of the feelings expressed by the carers is the relief. Nevertheless, Jacobson et al. [71] refers to the relief in relation to the burden of physical care, while our review reports other causes, such as having a nursing home placement offer [58]. In contrast, in relation to the support received, the study of Jacobson et al. [71] differs from ours in highlighting how carers normally did not receive support in an appropriate manner, neither from care providers, nor from the family, in order to continue with care at home, despite having sought this support.

Moreover, regarding negative experiences, family members highlight the feeling of concern, followed closely by the feelings of difficulty and conflict. These data from our results are consistent with a recent review on dementia in which, among other aspects, the decision-making of family carers is addressed [72]. The review of Livingston et al. [72] shows how families helping people with dementia highlight, that as representatives, making the decision on where that person should live or having to make plans for the person with dementia when their carer cannot continue to perform the care are some of the most difficult decisions to make [72].

With regard to the group of healthcare and social service professionals, although we have found few studies addressing their experiences in relation to this decision-making process, it is interesting to see how the results obtained do not differ too much from those obtained by other population groups. This can be found in our results in two main aspects. Firstly, the professionals report both positive and negative experiences, the latter being the most frequently reported and, secondly, within these negative experiences, the feeling most frequently reported was concern, similar to family members. However, it would be interesting to specifically address these experiences in future research on the subject, in order to understand in greater depth how professionals and other groups of relevant people involved in the decision experience this decision-making process.

Regarding the exclusive experiences of each population group found in this study, both the feelings of duty/responsibility and the feelings of exhaustion/feeling drained should be highlighted in the family members group. The latter is reflected in another review of the literature, which describes how admission to a facility is quite frequent because carers feel exhausted and no longer feel able to continue caring [71]. These are understandable and, in some ways, predictable situations, if we take into account that the people who usually perform the tasks of caring for the elderly are their closest relatives.

This review also indicates that although there are similar experiences that are evoked by the different population groups involved in the process, the participants often experience them differently, with different motivations or causations appearing in the different population groups. This is revealed in our results through different experiences, such as fear or concern. These experiences show how, despite having common elements, such as concern about costs, each population group experiences the process differently, focusing on the aspects that a priori seem to affect them the most. This highlights the importance of continuing to study this decision-making process from the points of view of the different people involved, because the perspectives of only one of the population groups studied will not be able to provide a complete understanding about the experience of this decision-making process for all involved.

Furthermore, it is interesting to highlight that this review is part of a broad review of the literature [11], which focused on the participants [12], motives [13], and experiences in decision-making process about the place of care for the elderly (informed review in this article). In these articles, valuable information has been obtained on each of these aspects, always providing a unique approach, which has taken into account the different types of participants involved in the process. This approach has helped us to understand in depth how this decision-making process takes place and the differences that exist between population groups. However, for future research it would be interesting to address this decision-making process for each of the possible places of care options, taking into account our multi-participant approach.

Finally, it is important to point out the importance of replicating this review in the coming years due to the health situation caused by the COVID-19 virus, which already has 88,828,387 confirmed cases and 1,926,625 deaths worldwide (as reported by the WHO as of 10 January 2021) [73]. Because of this, the authors of this review believe that in the coming years there will probably be an increase in scientific literature focused on the elderly and their care, since this population group is the one with the highest risk of severe complications, hospitalization, and death due to this virus [74]. Therefore, it would be extremely interesting for our review to be replicated once there are enough primary studies on this subject, in order to compare the experiences of the people involved in this decision-making process, taking into account the serious health crisis we have experienced. In doing so it would be possible to know if these experiences will continue or if a health crisis such as the one that COVID-19 has created will have a significant influence on them.

### Limitations

One of the most important limitations in the studies reviewed is that there is a lack of studies which focused on the experiences of professionals and other relevant people and this limits the understanding of the phenomenon from the perspective of all those involved.

In addition, it is important to highlight that on certain occasions the studies reviewed collected experiences reported by other direct participants of the process, but not by the person who directly experienced it (experiences that have been taken into account in the analysis), which implies a limitation in the understanding of the subjective and unique experience of the individual.

Regarding the limitations derived from the performance of this review, it should be taken into account that, due to the large amount of information analysed and the diversity in which the information was collected, in order to facilitate the synthesis work we decided to analyze the interpretations made by the authors of the included studies. However, certain quotations have also been synthesised and extracted on some occasions, due to their greater importance and precision for the purposes of this review.

Finally, due to the fact that the authors’ mother tongue is different from English, the classification, analysis, and interpretation of the results was conducted in Spanish, which has meant that the some of the experiences found have been translated and subsequently grouped under different categories simultaneously. However, it should be noted that, in these cases, a thorough reflexivity process has been performed to ensure consistency of the translation of these terms.

## 5. Conclusions

This review highlights a wide range of experiences, positive and negative, of those directly involved in the entire decision-making process on the place of care for the elderly.

These experiences have been studied in three main groups of the population involved: the elderly, family members, and professionals. A subsequent classification was made in each of these population groups, according to whether these experiences were positive or negative.

The negative experiences were expressed in a majority way in all the population groups studied. The number of negative experiences expressed was almost double that of the positive experiences mentioned by the participants.

Thus, in relation to the two most important population groups in our review, the elderly and family members, it is important to point out some aspects of interest. Within the group of the elderly, craving for the location, having previous experiences, and feeling support were the most relevant positive experiences. Conversely, the most frequent negative experiences are fear, concern, and difficulty.

With respect to family members, the feeling of professional and informal support and the feeling of relief were the most commonly mentioned positive experiences. While the concern, difficulty, and conflict were the most frequently mentioned negative experiences.

An important aspect found in this review has been the existence of experiences lived in a unique way by some of the population groups studied, as is the case of the elderly experiencing happiness or the family members feeling a sense of duty/responsibility. These experiences, despite being frequently commented on by these population groups, are not even mentioned by the rest of the population groups studied.

On the contrary, another aspect of interest in our review is the experiences expressed simultaneously by several population groups. However, these a priori common experiences, when studied in depth, are not experienced in the same way by the different population groups studied. This means that even though the experiences can be classified in the same way, it is very important to know what generates and provokes these experiences, since it varies according to the population group. Finally, we believe that more qualitative primary studies conducted with professionals and other relevant people involved in this decision-making process are needed, to know first-hand how they experience this process. Thus, achieving a much more complete theoretical corpus will facilitate the approach of the decision-making process to the services, professionals, and those affected by the decision.

## Figures and Tables

**Table 1:** Relevant positive experiences of the elderly ^a^.

Population: Type of Experience	Experience	Situations to Which This Experience Refers
Elders: positive experiences [23,24,25,26,27,28,29,30,31,32,33,34,35,36,37,38,39,40,41,42,43,44,45]	Crave for the location [24,25,30,31,33,36,39,45]	They were ready for it [24,25,30,31,33,36,39] Q1 [36]
		After having participated in the decision together with their family [45] Q2 [45]
	Previous experiences [25,27,28,29,30,31]	Due to experiences in relation to the care and/or relocation of a known relative [27,28,29,30,31] Q3 [30]
		Previous personal relocation experiences (of the elderly person himself/herself) [25]
	Feeling support [32,33,35,38,39]	From other people [32,35,38,39]: -Practical and emotional support from family and/or friends [32,35,38] -Professional support [32,38,39]
		For themselves [33,38]: -Their way of thinking [33] -Their way of being [38] -Their faith [38]

^a^ Source: own elaboration following the information obtained from the articles on which this systematic review is based.

**Table 2:** Relevant negative experiences of the elderly ^a^.

Population: Type of Experience	Experience	Situations to Which This Experience Refers
Elders: negative experiences [23,24,25,26,27,28,29,30,31,32,33,34,35,36,37,38,39,40,41,42,43,44,45,46,47,48,49,50,51,52,53,54]	Fear [24,27,28,30,32,34,39,42,49,51,52,53,54]	At the mere mention of the possibility of relocation [52]
		To their future [28]
		Of being a burden to their family [30] Q4 [30]
		Of having to rely on support or use places of care that they thought they could not afford [27] Q5 [27]
		Of the loss of autonomy/to dependence [42,51] Q6 [42]
		For their safety and other matters [34,39,49,54]: With special attention to falls [39,49]
		Of isolation/loneliness [32,53] Q7 [32]
	Concern [23,25,27,31,34,39,40,41,42,48,51]	For their future [27,31]
		For the home itself [27,34]
		For relocation [39,41]
		For the new place of care [51]
		For the organization/costs [23,27,40,42]
		For their family [27,48]
		For losing autonomy/for dependence [25,27,40]
		With regards to their safety [34,39]
	Difficulty, in relation to several aspects. [24,25,27,30,35,39,40,43,44,54]	To the relocation [24,30,39,54] Q8 [24]
		To their home [24,30,43,44] Q9 [24]
		To the organization and costs [27,40]
		To the little time within which the decision was made [35]
		Not knowing what to choose [25,27]
		To the stigmas associated with age [43]
		Being a problematic decision [27,43]

^a^ Source: own elaboration following the information obtained from the articles on which this systematic review is based.

**Table 3:** Relevant positive experiences of family members ^a^.

Population: Type of Experience	Experience	Situations to Which This Experience Refers
Family: positive experiences [23,25,26,32,34,35,36,37,40,47,52,54,55,56,57,58,59,60,61,62,63]	The support of the professionals [26,37,47,52,55]	In the care [37,55]
		With positive interactions and/or feelings between family and professionals [37,47,52,55]
		Taking care of family dynamics, improving communication between family members [47]
		Regarding the operation of the system [37,47]
		In the decision itself [26,37,47,55] Q10 [47]
	Informal network support [26,55,56,61,62]	In the care experience [55] Q11 [55]
		Helping to confirm if the current environment meets the needs of the elderly person [26]
		In the search for help and solutions [61]
		Being supported in their decision by other relatives [62]
		Validating the decisions made recognizing the appropriateness of the decision [56] Q12 [56]
	Relief [37,57,58,62]	By keeping the elderly person at home by activating external help [37]
		For having a nursing home placement offer [58]
		For family support for the decision [62]
		For the admission decision [57]

^a^ Source: own elaboration following the information obtained from the articles on which this systematic review is based.

**Table 4:** Relevant negative experiences of family members ^a^.

Population: Type of Experience	Experience	Situations to Which This Experience Refers
Family: negative experiences [23,24,25,26,27,30,32,34,35,36,37,38,39,47,49,50,52,54,55,56,57,58,59,60,61,62,63,64,65,66]	Concern [23,24,25,26,30,35,36,38,39,47,49,50,55,57,58,59,60,61,62,63,64,65,66]	About the decision of their elderly people to relocate [25]
		About the relocation process [38]
		About the elder [24,26,30,36,39,47,50,55,58,60,62,63,65]
		About the carer’s situation [26,49,58,62,66]
		About the family [62,64]
		About others who face the location situation [63] Q13 [63]
		About the different costs [23,35,57,58,61,62,63,64,65] Q14 [35]
		About the opinions of others [62,64] Q15 [62]
		About the new location of the elderly person [57,58,60,63,64]
		About other people (outside the family) caring for the elder [49,59] Q16 [49]
	Difficulty [24,26,37,47,49,52,55,56,57,59,60,61,62,63,64,65,66]	Regarding the care [24,26,49,56,59,60,61,62,66]
		Because the older person does not see the need for relocation [55] Q17 [55]
		Because the carers do not understand what was happening [57]
		In accepting the need for location [56,57] Q18 [56]
		Due to lack of time in the decision-making process [56,63]
		With the fact that it is a difficult decision [37,47,64,65]
		In the decision making itself [57,64]
		In deciding for the elderly person and/or feeling responsible for the elderly person [26,52]
		In incorporating the values of the elderly in the decision making [65]
		In balancing the needs of those involved [62]
		Because of family dynamics [47,62,63,64]
		In navigating through the health system (if collaboration with professionals is not optimal) [47]
		Due to lack of information and financial concerns [57]
		In related matters: how to sell/get rid of the elderly person’s house, or due to transportation [57]
		In finding the right place [63]
		In the placement process itself [57,60,63]
	Conflict [32,34,50,54,57,59,60,61,62,63,64,65,66]	Due to the safety and independence of the elderly [34,60] Q19 [60]
		Within the family [50,57,62,63,64,65] Q20 [50]
		With the marriage vows [61]
		With the care [54]
		With the expectations of society [66]
		Between the needs of the elderly and their own needs [34,59,65,66] Q21 [66]
		With other responsibilities [32,59,62]

^a^ Source: own elaboration following the information obtained from the articles on which this systematic review is based.

**Table 5:** Unique relevant experiences by population group ^a^.

Population: Type of Experience	Experience	Situations to Which This Experience Refers
Elders: unique positive experience	Happiness [30,37,39,40]	Because of how the location process was developed [39]
		When talking about having exchanged space for security reasons [40]
		For being able to take their pet to the new place of care [37]
Family: unique negative experience	Duty/Responsibility [34,58,59,60,61,62,63]	To keep the elderly person at home while possible [58]
		Feel care/attention as a duty or responsibility [34,59,60,61,62] Q22 [59]
		Feeling of responsibility for the elderly [63]
	Exhaustion/feeling drained [26,32,37,47,54,55,57,58,59,61,62,64]	For reasons related to the care of the elderly [26,32,37,47,54,55,57,58,59,61,62,64] Q23 [55], Q24 [47], Q25 [26], Q26 [61]: -Making them feel unable to continue caring due to the harmful effects of care on their own health, family life, or employment [57].

^a^ Source: own elaboration following the information obtained from the articles on which this systematic review is based.

## Supplementary Materials

The following are available online at https://www.mdpi.com/2076-328X/11/2/14/s1, This article has four Supplementary Materials: The first is the PRISMA checklist (Table S1: PRISMA Checklist); the second shows a table with the characteristics of the included studies (Table S2: Characteristics of the included studies); the third shows different summary tables with all the experiences found in our review (Table S3: List of experiences); the fourth is a document that shows textual quotes that support our results (Text S4: Original Quotes).

## References

[1] (2019). World Population Prospects 2019: Highlights. https://population.un.org/wpp/Publications/Files/WPP2019_Highlights.pdf.

[2] Organización Mundial de la Salud (2015). Informe Mundial sobre el envejecimiento y la salud. https://apps.who.int/iris/bitstream/handle/10665/186466/9789240694873_spa.pdf;jsessionid=54558A8AD04FB1D596ED8222A0EF33D3?sequence=1.

[3] (2003). The Future Supply of Long-Term Care Workers In Relation to the Aging Baby Boom Generation: Report To Congress. https://aspe.hhs.gov/system/files/pdf/72961/ltcwork.pdf.

[4] Kogan A.C., Wilber K., Mosqueda L. (2016). Person-Centered Care for Older Adults with Chronic Conditions and Functional Impairment: A Systematic Literature Review. J. Am. Geriatr. Soc..

[5] United Nations Humans Rigths Office of the High Commissioner (1991). United Nations Principles for Older Persons. https://www.ohchr.org/EN/ProfessionalInterest/Pages/OlderPersons.aspx.

[6] Schumacher K.L., Meleis A.l., Meleis A. (2009). Theoretical development of transitions: Transitions: A central concept in nursing. Transitions Theory: Middle-range and Situation-specific Theories in Nursing Research and Practice.

[7] Hays J.C. (2002). Living arrangements and health status in later life: A review of recent literature. Public Health Nurs..

[8] Oswald F., Rowles G.D., Wahl H.-W., Tesch-Römer C., Hoff A. (2017). Beyond the relocation trauma in old age: New trends in elders’ residential decisions. New Dynamics in Old Age: Individual, Environmental and Societal Perspectives.

[9] Bandyopadhyay D., Pammi V.S.C., Srinivasan N., Pammi V.S.C., Srinivasan N. (2013). Chapter 3—Role of affect in decision making. Progress in Brain Research.

[10] King L., Harrington A., Linedale E., Tanner E. (2018). A mixed methods thematic review: Health-related decision-making by the older person. J. Clin. Nurs..

[11] Serrano-Gemes G., Serrano-del-Rosal R., Rich-Ruiz M. (2018). Decision-making on the location of care of the elderly: Protocol for a systematic review of qualitative studies. BMJ Open.

[12] Serrano-Gemes G., Rich-Ruiz M., Serrano-del-Rosal R. (2020). Systematic review of qualitative studies on participants in the decision-making process about the location of care of the elderly. BMJ Open.

[13] Serrano-Gemes G., Rich-Ruiz M., Serrano-del-Rosal R. (2020). Reasons for the Place of Care of the Elders: A Systematic Review. Healthcare.

[14] Tong A., Flemming K., McInnes E., Oliver S., Craig J. (2012). Enhancing transparency in reporting the synthesis of qualitative research: ENTREQ. BMC Med. Res. Methodol..

[15] Moher D., Liberati A., Tetzlaff J., Altman D.G., PRISMA Group (2009). Preferred reporting items for systematic reviews and meta-analyses: The PRISMA statement. PLoS Med..

[16] Glaser B.G. (1965). The Constant Comparative Method of Qualitative Analysis. Soc. Probl..

[17] Glaser B.G., Strauss A.L. (1967). The Discovery of Grounded Theory: Strategies for Qualitative Research.

[18] Lockwood C., Porritt K., Munn Z., Rittenmeyer L., Salmond S., Bjerrum M., Loveday H., Carrier J., Stannard D., Aromataris E., Munn Z. (2020). Chapter 2: Systematic reviews of qualitative evidence. JBI Manual for Evidence Synthesis.

[19] Thomas J., Harden A. (2008). Methods for the thematic synthesis of qualitative research in systematic reviews. BMC Med. Res. Methodol..

[20] Cano Arana A., González Gil T., Cabello López J. (2010). CASPe. Plantilla para ayudarte a entender un estudio cualitativo. CASPe. Guías CASPe de Lectura Crítica de la Literatura Médica.

[21] Butler A., Hall H., Copnell B. (2016). A Guide to Writing a Qualitative Systematic Review Protocol to Enhance Evidence-Based Practice in Nursing and Health Care. Worldviews Evid. Based Nurs..

[22] Dixon-Woods M., Bonas S., Booth A., Jones D.R., Miller T., Sutton A.J., Shaw R.L., Smith J.A., Young B. (2006). How can systematic reviews incorporate qualitative research? A critical perspective. Qual. Res..

[23] Ayalon L. (2016). Intergenerational perspectives on autonomy following a transition to a continuing care retirement community. Res. Aging.

[24] Bekhet A.K., Zauszniewski J.A., Nakhla W.E. (2009). Reasons for relocation to retirement communities: A qualitative study. West. J. Nurs. Res..

[25] Cheng Y., Rosenberg M.W., Wang W., Yang L., Li H. (2012). Access to residential care in Beijing, China: Making the decision to relocate to a residential care facility. Ageing Soc..

[26] Ducharme F., Couture M., Lamontagne J. (2012). Decision-making process of family caregivers regarding placement of a cognitively impaired elderly relative. Home Health Care Serv. Q..

[27] Gabrielsson-Jarhult F., Nilsen P. (2016). On the threshold: Older people’s concerns about needs after discharge from hospital. Scand. J. Caring Sci..

[28] Gottlieb A.S., Stoeckel K.J., Caro F.G. (2009). Residential adjustment of elders: Learning from experiences with parents and peers. J. Hous. Elder..

[29] Groger L. (1994). Decision as process: A conceptual model of Black elders’ nursing home placement. J. Aging Stud..

[30] Groger L., Kinney J. (2006). CCRC here we come! Reasons for moving to continuing care retirement community. J. Hous. Elder..

[31] Hartwigsen G. (1987). Older widows and the transference of home. Int. J. Aging Hum. Dev..

[32] Heppenstall C.P., Keeling S., Hanger H.C., Wilkinson T.J. (2014). Perceived factors which shape decision-making around the time of residential care admission in older adults: A qualitative study. Australas. J. Ageing.

[33] Iwasiw C., Goldenberg D., MacMaster E., McCutcheon S., Bol N. (1996). Residents’ perspectives of their first 2 weeks in a long-term care facility. J. Clin. Nurs..

[34] Jenkins C.L. (2003). Care arrangement choices for older widows: Decision participants’ perspectives. J. Women Aging.

[35] Kemp C.L. (2008). Negotiating transitions in later life: Married couples in assisted living. J. Appl. Gerontol..

[36] Laditka S.B. (2017). «It Can’t Happen Soon Enough.» The Role of Readiness in Residential Moves by Older Parents. Gerontologist.

[37] Lynch J.S. (2006). The cycle of relocation: One family’s experience with elder care. Top. Adv. Pract. Nurs. eJ..

[38] McKenna D., Staniforth B. (2017). Older people moving to residential care in Aotearoa New Zealand: Considerations for social work at practice and policy levels. Aotearoa N. Z. Soc. Work.

[39] Nord C. (2016). Free choice in residential care for older people—A philosophical reflection. J. Aging Stud..

[40] Peace S., Holland C., Kellaher L. (2011). «Option recognition» in later life: Variations in ageing in place. Ageing Soc..

[41] Saunders J.C., Heliker D. (2008). Lessons learned from 5 women as they transition into assisted living. Geriatr. Nurs..

[42] Söderberg M., Ståhl A., Melin Emilsson U. (2013). Independence as a stigmatizing value for older people considering relocation to a residential home. Eur. J. Soc. Work.

[43] Vasara P. (2015). Not ageing in place: Negotiating meanings of residency in age-related housing. J. Aging Stud..

[44] Walker E., McNamara B. (2013). Relocating to retirement living: An occupational perspective on successful transitions. Aust. Occup. Ther. J..

[45] Wilson D.M., Vihos J., Hewitt J.A., Barnes N., Peterson K., Magnus R. (2013). Examining waiting placement in hospital: Utilization and the lived experience. Glob. J. Health Sci..

[46] Chen S., Brown J.W., Mefford L.C., de La Roche A., McLain A.M., Haun M.W., Persell D.J. (2008). Elders’ decisions to enter assisted living facilities: A grounded theory study. J. Hous. Elder..

[47] Couture M., Ducharme F., Lamontagne J. (2012). The Role of Health care Professionals in the Decision-Making Process of Family Caregivers Regarding Placement of a Cognitively Impaired Elderly Relative. Home Health Care Manag. Pract..

[48] Ewen H.H., Chahal J. (2013). Influence of Late Life Stressors on the Decisions of Older Women to Relocate into Congregate Senior Housing. J. Hous. Elder..

[49] Jorgensen D., Arksey H., Parsons M., Senior H., Thomas D. (2009). Why do older people in New Zealand enter residential care rather than choosing to remain at home, and who makes that decision?. Ageing Int..

[50] Koenig T.L., Lee J.H., Macmillan K.R., Fields N.L., Spano R. (2014). Older adult and family member perspectives of the decision-making process involved in moving to assisted living. Qual. Soc. Work.

[51] Löfqvist C., Granbom M., Himmelsbach I., Iwarsson S., Oswald F., Haak M. (2013). Voices on relocation and aging in place in very old age—A complex and ambivalent matter. Gerontologist.

[52] Söderberg M., Ståhl A., Melin Emilsson U. (2012). Family members’ strategies when their elderly relatives consider relocation to a residential home—Adapting, representing and avoiding. J. Aging Stud..

[53] Tyvimaa T., Kemp C.L. (2011). Finnish Seniors’ Move to a Senior House: Examining the Push and Pull Factors. J. Hous. Elder..

[54] Vassallo T. (1995). Systemic therapy and aged respite care: A neglected area. Aust. N. Z. J. Fam. Ther..

[55] Caron C.D., Ducharme F., Griffith J. (2006). Deciding on institutionalization for a relative with dementia: The most difficult decision for caregivers. Can. J. Aging Rev. Can. Vieil..

[56] Dellasega C., Mastrian K. (1995). The process and consequences of institutionalizing an elder. West. J. Nurs. Res..

[57] Dellasega C., Nolan M. (1997). Admission to care: Facilitating role transition amongst family carers. J. Clin. Nurs..

[58] Fjelltun A.-M.S., Henriksen N., Norberg A., Gilje F., Normann H.K. (2009). Carers’ and nurses’ appraisals of needs of nursing home placement for frail older in Norway. J. Clin. Nurs..

[59] Kao H.F., Stuifbergen A.K. (1999). Family experiences related to the decision to institutionalize an elderly member in Taiwan: An exploratory study. Soc. Sci. Med..

[60] Koplow S.M., Gallo A.M., Knafl K.A., Vincent C., Paun O., Gruss V. (2015). Family Caregivers Define and Manage the Nursing Home Placement Process. J. Fam. Nurs..

[61] Mamier I., Winslow B.W. (2014). Divergent views of placement decision-making: A qualitative case study. Issues Ment. Health Nurs..

[62] Park M., Butcher H.K., Maas M.L. (2004). A thematic analysis of Korean family caregivers’ experiences in making the decision to place a family member with dementia in a long-term care facility. Res. Nurs. Health.

[63] Rodgers B.L. (1997). Family members’ experiences with the nursing home placement of an older adult. Appl. Nurs. Res..

[64] Chang Y.-P., Schneider J.K. (2010). Decision-making process of nursing home placement among Chinese family caregivers. Perspect. Psychiatr. Care.

[65] Légaré F., Stacey D., Brière N., Robitaille H., Lord M.-C., Desroches S., Drolet R. (2014). An interprofessional approach to shared decision making: An exploratory case study with family caregivers of one IP home care team. Bmc Geriatr..

[66] Tamiya N., Chen L.-M., Sugisawa H. (2009). Caregivers’ decisions on placement of family members in long-term care facilities in Japan: Analysis of caregiver interviews. Soc. Behav. Pers..

[67] Lee D.T.F., Woo J., Mackenzie A.E. (2002). A review of older people’s experiences with residential care placement. J. Adv. Nurs..

[68] Fjordside S., Morville A. (2016). Factors influencing older people’s experiences of participation in autonomous decisions concerning their daily care in their own homes: A review of the literature. Int. J. Older People Nurs..

[69] Rossen E.K., Knafl K.A. (2007). Women’s Well-Being After Relocation to Independent Living Communities. West. J. Nurs. Res..

[70] Maust D.T., Blass D.M., Black B.S., Rabins P.V. (2008). Treatment decisions regarding hospitalization and surgery for nursing home residents with advanced dementia: The CareAD Study. Int. Psychogeriatr..

[71] Jacobson J., Gomersall J.S., Campbell J., Hughes M. (2015). Carers’ experiences when the person for whom they have been caring enters a residential aged care facility permanently: A systematic review. JBI Database System Rev. Implement. Rep..

[72] Livingston G., Sommerlad A., Orgeta V., Costafreda S.G., Huntley J., Ames D., Ballard C., Banerjee S., Burns A., Cohen-Mansfield J. (2017). Dementia prevention, intervention, and care. Lancet.

[73] World Health Organization WHO Coronavirus Disease (COVID-19) Dashboard. [Data last updated: 10/01/2021, 10:14am CET]. https://covid19.who.int/.

[74] CDC Centers for Disease Control and Prevention Your Health: Older Adults. [Data last updated: 13/12/2020]. https://www.cdc.gov/coronavirus/2019-ncov/need-extra-precautions/older-adults.html#:~:text=The%20risk%20for%20severe%20illness,older%20adults%20at%20highest%20risk.&text=need%20to%20know-,Risk%20for%20severe%20illness%20with%20COVID%2D19%20increases%20with%20age,increase%20risk%20for%20severe%20illness.

